# Protection against lethal HAdV-4 challenge in STAT1 mice by novel human monoclonal antibodies

**DOI:** 10.3389/fimmu.2025.1613945

**Published:** 2025-06-19

**Authors:** Xinyi Zhang, Zhongge Zhu, Peijie Zhai, Peng Lv, You Yang, Chuanhang Cheng, Busen Wang, Ting Fang, Guanying Zhang, Xiangyang Chi, Jianmin Li, Wei Chen, Yunzhu Dong

**Affiliations:** ^1^ College of Pharmacy, Nanjing University of Chinese Medicine, Nanjing, China; ^2^ Laboratory of Advanced Biotechnology, Beijing Institute of Biotechnology, Beijing, China; ^3^ College of Acupuncture, Moxibustion and Tuina, Nanjing University of Chinese Medicine, Nanjing, China

**Keywords:** human adenovirus serotype 4, neutralizing monoclonal antibody, stat1^-/- ^transgenic mice, structural biology, TRIM21

## Abstract

**Introduction:**

Human adenovirus serotype 4 (HAdV-4) is an epidemic pathogen associated with severe acute respiratory disease (ARD) in both pediatric and adult populations. Currently, no available vaccine or therapeutic interventions specifically targeting adenoviruses are available.

**Methods:**

In this study, we isolated peripheral blood mononuclear cells (PBMCs) from HAdV-4 infected donors and generated fully human monoclonal antibodies using single-cell PCR technology. The antibodies were first characterized for their neutralization efficacy both in vitro and in vivo. Subsequently, we predicted key functional residues through structural modeling of antigen-antibody complexes and validated their roles via mutagenesis studies. Finally, the mechanism of intracellular neutralization of antibodies was explored.

**Results:**

Through systematic screening, we successfully isolated seven antibodies with specific binding activity, among which monoclonal antibodies (mAbs) 2CF4 and 4AC3 exhibited potent neutralizing capacity against HAdV-4. Notably, we modeled adenoviral lethality using Stat1^-/-^ transgenic mice, mAb 2CF4 conferred full protection against HAdV-4 infection in Stat1^-/-^ transgenic mice. We identified critical amino acid residues, R99, R102 and T104 aa, of mAb 2CF4 by structural prediction of the antigen-antibody complex. Furthermore, the mAb 2CF4 neutralize the HAdV-4 through the interaction with the widely expressed cytoplasmic Fc-binding protein TRIM21.

**Discussion:**

Overall, mAb 2CF4 represents a promising candidate for safe and effective prophylactic and therapeutic strategies against HAdV-4 infection.

## 1 Introduction

Significant outbreaks attributed to HAdV-4 have been documented, underscoring a rising morbidity associated with this virus in various countries (1–4). Recent Outbreaks of severe acute febrile respiratory disease and pneumonia attributed to HAdV-4 infections have occurred among pediatric populations in India, Taiwan, and South Korea (5–8) as well as in healthy immunocompetent adults in the United States, Italy, and Singapore (3, 9). In Beijing, a notable outbreak involved over 50 individuals, including teachers and staff at a swimming pool, with at least one reported instance of probable person-to-person transmission (10). Alarmingly, a total of 27 fatalities have been reported, with 26 attributed to respiratory complications and one resulting from encephalitis following respiratory illness (11).

HAdV is classified as a non-envelope, icosahedral, double-stranded DNA virus within the Mastadenovirus genus of the Adenoviridae family, which encompasses seven species (A-G) (12–15). HAdV-4 is the sole representative of species E, with respiratory and ocular diseases being the most common manifestations of infection. The surface of HAdV consists of three structural proteins: Fiber, Penton and Hexon, of which Hexon is the main structural protein of adenovirus, containing 936 hydroxyl amino acids, and contains a large number of specific antigenic epitopes on its surface, which encode the major structural elements involved in the antigen-antibody binding of HAdV (16).

Among all adenovirus types, HAdV-4 and HAdV-7 have been identified as the principal etiological agents of recurrent acute respiratory disease (ARD) and pneumonia in recruits of all United states military services (17–19). Following the FDA’s approval of oral live viral vaccines for HAdV-4 and HAdV-7 in 2011, the incidence of adenovirus-related diseases among military recruits decreased by approximately 100-fold over the subsequent two years (20–22). Currently, in the United States, the live oral HAdV-4 and HAdV-7 vaccine remains exclusively approved for military personnel, while no comparable vaccine development programs exist in China. Currently, the management of human adenovirus infections primarily relies on symptomatic supportive therapy and antiviral treatment, including rest, rehydration, antipyretics, ribavirin, intravenous immunoglobulin (IVIG), and cytokine therapy. Furthermore, no targeted antiviral therapies have been approved specifically for HAdV-4 infection. Consequently, there is an urgent need for effective therapeutic strategies against HAdV-4 infections. Neutralizing antibodies against viruses represent a promising strategy for the prevention and treatment of viral infections (23). The primary structural proteins of adenoviruses include Hexon, Penton and Fiber. Neutralizing antibodies against adenoviruses can be generated against any of these major capsid proteins (24, 25), however the Hexon protein is the predominant target of serotype-specific neutralizing antibodies (nAbs) (26–30). Currently, only mouse anti-humanized antibodies have been isolated (31), and no monoclonal antibodies specific to HAdV-4 have been isolated from human sources.

The signal transducer and activator of transcription STAT1 protein plays a key role in the immune response against viruses and other pathogens by transducing, in the nucleus, the signal from type I, type II and type III IFNs. STAT1 gene deletion in mice and complete STAT1 deficiency in humans result in severe susceptibility to infections, often leading to rapid mortality (32). Given its critical role, we employed Stat1^-/-^ mice as an animal model to evaluate the protective efficacy of antibodies *in vivo*.

In this study, we identified two human monoclonal antibodies, 2CF4 and 4AC3, targeting the Hexon protein of HAdV-4. MAb 2CF4 specifically recognized conformational epitope of HAdV-4 and exhibited potent neutralizing activity *in vitro*. To further assess their therapeutic potential, we established a lethal adenovirus infection model in mice and demonstrated that 2CF4 effectively neutralized the virus at low concentrations, providing significant protection *in vivo*. To unravel the molecular basis of neutralization, we predicted antigen-antibody complexes to identify critical amino acid residues involved in binding. The functional importance of these residues was subsequently validated through alanine-scanning mutagenesis. Additionally, tripartite motif-containing protein 21 (TRIM21), a member of the TRIM family with E3 ubiquitin ligase activity, has been implicated in viral infections through direct interactions with viral proteins or modulation of immune and inflammatory responses. TRIM21 also functions as a high-affinity cytosolic Fc receptor, binding to antibody-virus complexes and initiating antibody-dependent intracellular neutralization (ADIN) (33). To explore this mechanism, we employed immunofluorescence assays to visualize intracellular fluorescence, confirming the involvement of TRIM21 in the intracellular neutralization process.

## 2 Materials and methods

### 2.1 Viruses and cell lines

A549 and HEK293 cells were maintained in Dulbecco’s Modified Eagle’s Medium (DMEM, Gibco) supplemented with 10% fetal bovine serum (FBS, Gibco) and 2% Penicillin-Streptomycin. Expi293F cells were cultured in FreeStyle™ 293 Expression Medium (Gibco). All cells were incubated at 37°C in a humidified atmosphere with 5% CO_2._ The Ad4-RI67 and Ad4-Luc recombinant virus used in this study were generated in our laboratory.

### 2.2 Expression and purification of the Hexon protein

HAdV-4 infected 293F cells were harvested after 72 h, followed by centrifugation and three freeze-thaw cycles at -80°C. The lysate was then purified using a Core 700 molecular sieve (Cytiva) with a buffer containing 20 mM Tris and 150 mM NaCl (pH 7.5). Subsequently, the protein was concentrated using an ultrafiltration tube and further purified by size exclusion chromatography on a Superdex 200 molecular sieve (Cytiva).

### 2.3 Antigen-specific B cell sorting

PBMCs were isolated from three Chinese convalescent patients who had naturally contracted HAdV-4 infection using Ficoll density gradient centrifugation. The PBMCs were stained with fluorescently labeled antibodies, including anti-IgG-PE (555787, BD), anti-CD19-Alexa Fluor 700 (302226, Biolegend), anti-CD27-PE-Cy7 (560609, BD), and anti-CD3-PerCP (300326, Biolegend), purified HAdV-4 Hexon proteins and tagged them with biotin, and PE-streptavidin (405203, Biolegend), at 4°C for 1 h. After staining, the cells were washed twice with cell staining buffer. B cells were then sorted using a MA900 cell sorter (SONY), with target cells defined as CD3^−^CD19^+^IgG^+^CD27^+^streptavidin^+^ (Hexon). Single cells were sorted into 96-well plates pre-filled with RNase-free water and RNase inhibitor (Promega). The plates were flash-frozen in liquid nitrogen and subsequently stored at −80°C for further analysis.

### 2.4 Antibody preparation

Double-stranded cDNAs encoding the variable heavy (VH) and light (VL) chain genes of antibodies were synthesized directly in the sorted-cell plates using a One-Step RT-PCR kit (Qiagen), followed by nested PCR amplification with TransStart Taq DNA polymerase (TransGen Biotech) as previously described. The resulting PCR products were sequenced by Sangon Biotech. For rapid antibody expression, linear expression cassettes were employed. Specifically, overlapping extension PCR was performed using CMV-UP and TK-POLYA as primers to amplify the linear heavy (H), kappa (κ), and lambda (λ) chains, with the promoter-precursor fragment, constant region-polyA tail fragment, and variable region fragment serving as templates. The full-length antibody sequences were subsequently cloned into the pcDNA3.4 vector. Paired heavy and light chain plasmids were co-transfected into Expi293F cells (Thermo Fisher Scientific) following the manufacturer’s instructions. Finally, antibodies were purified from cell culture supernatants using a HiTrap rProtein A column (Cytiva).

### 2.5 Enzyme-linked immunosorbent assay

Purified Hexon protein (2 μg/mL) was coated onto ELISA microplates (Corning) and incubated overnight at 4°C. After washing with PBST (PBS containing 0.2% Tween-20), the plates were blocked with 2% bovine serum albumin (BSA; Sigma-Aldrich) in PBST for 1 h at 37°C. Following another wash with PBST, serially diluted test antibodies were added to each well and incubated at 37°C for 1 h. The plates were then washed again, and horseradish peroxidase (HRP)-conjugated anti-human IgG antibody (Abcam) was added at a 1:10,000 dilution, followed by incubation at 37°C for 1 h. After a final wash, TMB single-component substrate solution (Solarbio) was added to the wells and incubated at room temperature for 6 min. The reaction was stopped by adding 2 M H_2_SO_4_, and the absorbance was measured at 450 nm with a reference wavelength of 630 nm.

### 2.6 Competition binding analysis

Detection antibodies were biotinylated using EZ-Link™ Sulfo-NHS-Biotin (Thermo Scientific) according to the manufacturer’s instructions. Microplates were coated with 2 μg/mL Hexon protein and incubated overnight at 4°C. A mixture of detection antibodies (final concentration: 10 ng/mL) and blocking antibodies (final concentration: 1 μg/mL) was added to each well and incubated for 1 h at 37°C. After washing with PBST, streptavidin conjugated with horseradish peroxidase (HRP; Thermo Scientific) was added at a concentration of 1 μg/mL and incubated for 1 h at 37°C. Following another wash with PBST, TMB single-component substrate solution (Solarbio) was added and incubated at room temperature for 6 min. The reaction was stopped by adding 2 M H_2_SO_4_, and the absorbance was measured at 450 nm with a reference wavelength of 630 nm. The competition value was calculated as the ratio of the binding signal of the detection antibody in the presence of the blocking antibody to the binding signal of the detection antibody alone.

### 2.7 Monoclonal antibody neutralization experiment against HAdV-4

To assess the neutralization efficacy of HAdV-4-specific antibodies against HAdV-4, 50 μL of the Ad4-Luc recombinant virus was mixed with 50 μL of a monoclonal antibody (initial concentration: 10 μg/mL) in 96-well cell culture plates and incubated at 37°C for 1 h. Subsequently, 2 × 10^5^ A549 cells suspended in 100 μL of DMEM supplemented with 10% FBS were added to each well. The plates were then incubated at 37°C in 5% CO_2_ for 24 h. After incubation, 200 μL of the medium was carefully removed, and 100 μL of Luciferase Assay Cell Culture Lysis 5× Reagent was added to each well, followed by a 10 min incubation at 37°C. The lysate was mixed 10 times by pipetting, and 20 μL of the mixture was transferred to a white opaque plate. Luciferase activity was measured using a microplate luminometer (Promega).

### 2.8 Surface plasmon resonance assay

Antibody-antigen binding kinetics were analyzed using surface plasmon resonance (SPR) technology on a Biacore T200 instrument (GE Healthcare). The antibody was diluted to a concentration of 0.5 μg/mL in HBS-EP+ buffer (GE Healthcare) and captured on a Protein A chip at a flow rate of 10 μL/min for 60 s. The purified antigen was tested at serially diluted concentrations (100, 50, 25, 12.5, and 6.25 nM) with a flow rate of 30 μL/min. The association phase was monitored for 120 s, followed by a 600 s dissociation phase.

### 2.9 Authentic virus-neutralizing assay

All experiments involving authentic HAdV-4 in this study were conducted in a biosafety level 2 (BSL-2) facility. The neutralizing activity of antibodies against the wild-type HAdV-4 strain (RI67) was evaluated using a microneutralization assay, as previously described. Briefly, 3 × 10^4^ A549 cells in 100 μL of DMEM supplemented with 10% FBS were seeded into each well of 96-well cell culture plates and incubated overnight at 37°C with 5% CO_2_. Antibodies (initial concentration: 100 μg/mL) were serially diluted three-fold in 100 μL volumes and mixed with an equal volume of 100 TCID_50_ HAdV-4 in sterile 96-well plates, followed by incubation at 37°C for 1 h. Following neutralization, 100 μL of each antibody-virus mixture was carefully transferred onto confluent A549 cell, followed by incubation at 37°C for 1 h. Following removing the supernatants, 200 µL cell culture medium were added and the plates were then incubated at 37°C with 5% CO_2_ for 3 days. The infected cells were observed under a microscope, and cell viability was assessed using a Cell Counting Kit-8 (CCK-8) assay (Vazyme Biotech) according to the manufacturer’s protocol. The 96-well plates were washed twice with sterile PBS, and 100 μL of DMEM medium containing 2% FBS and 10% CCK-8 reagent was added to each well. The plates were then incubated at 37°C for 30 min, and the absorbance at 450 nm was measured using a microplate reader. 

### 2.10 *In vivo* animal challenge experiment

Stat1^-/-^ transgenic mice (Shanghai Model Organisms Center), aged 6–8 weeks, were housed in a biosafety level-2 (BSL-2) facility and provided with ad libitum access to standard pellet feed and water. All infectious experiments were conducted in compliance with the standard operating procedures of the approved BSL-2 facility and were approved by the Animal Experiment Committee of the Laboratory Animal Center, Academy of Military Medical Sciences. Mice were intraperitoneally injected with 3 × 10^10^ PFUs of Ad4-RI67. Mice in the prophylactic groups were administered 2CF4 (50 μg, 10 μg, or 2 μg) one day prior to infection. Body weights and survival rates were monitored daily for 14 days post-infection. Additionally, lung and liver tissues were collected at 4 days post-infection to assess changes in viral replication. Genomic DNA of HAdV-4 was extracted from tissues using the QIAamp DNA Mini Kit (Qiagen) and quantified by RT-qPCR using SYBR Green Supermix (Thermo Fisher). The primers used were as follows: HAdV-4-F, 5’-CAAGGACTACCAGGCCGTCA-3’ and HAdV-4-R, 5’-GTTAGCATAGAGCATGTTCT-3’. The RT-qPCR program consisted of an initial denaturation at 50°C for 2 min, 95°C for 10 min, followed by 40 cycles of 95°C for 15 s and 60°C for 1 min. Following complete annealing of all single-stranded DNA at 60°C, the temperature was gradually increased to 95°C at a ramp rate of 0.15°C/s, inducing progressive dissociation of the double-stranded DNA. Fluorescence signal variations during this denaturation process were detected by the instrument to generate the melting curve profile. Viral genome copies were quantified using standard curves generated from serially diluted genomic DNA extracted from viral stocks.

### 2.11 AlphaFold mimic antigen-antibody complexes

The amino acid sequence of the Hexon protein was obtained from GENBANK: AAT97450.1. The heavy chain and light chain sequences of the mAb 2CF4, along with the Hexon sequence, were used as input for AlphaFold 2.3.2. The multimer mode of AlphaFold 2.3.2 was used to predict protein complexes, resulting in the structural model of the antibody 2CF4 complexed with the Hexon protein monomer. Default parameters were used during the prediction process, and the confidence of the resulting models was evaluated using the predicted Local Distance Difference Test (pLDDT) score, with the highest-scoring models selected for further analysis. The trimeric structure of Hexon (PDB ID: 2OBE) was used as a template. Using the “Structure Analysis/Matchmaker” Tool in ChimeraX 1.2.4, the predicted model was repeatedly overlapped with the template structure, resulting in a simulated structure of the antibody 2CF4 complexed with the trimeric Hexon protein.

### 2.12 Immunofluorescence

A549 cells (2 × 10^5^) were seeded onto coverslips in 24-well plates and allowed to adhere overnight. The cells were washed twice with DMEM prior to infection. Ad4-RI67 (1 × 10^7^ PFUs) was pre-incubated with an adenovirus-neutralizing antibody (10 μg) in 500 μL of DMEM for 30 min at room temperature. The cells were then infected with 500 μL of this mixture for 6 h at 37°C. After infection, the cells were washed three times with PBS, fixed with 4% paraformaldehyde for 20 min, permeabilized with 0.5% Triton X-100 in PBS for 15 min, and blocked with PBS containing 5% BSA and 0.1% Tween-20 (PBS-BSA) for 1 h. Immunostaining was performed using a rabbit anti-TRIM21 antibody (12108-1-AP, Proteintech) and a goat anti-ubiquitin antibody (97003, Abcam), both diluted 1:200 in PBS-BSA. Alexa Fluor 594-conjugated secondary antibodies were used at a 1:1000 dilution to detect the primary antibodies. Additionally, an anti-E-cadherin antibody (Abcam) was used to stain cell membranes. Confocal images were acquired using a Zeiss 63× objective lens on an Axio Observer microscope.

### 2.13 Generation of stable knockdown and overexpressing cell lines

The TRIM21 gene was cloned into the pLVX-C-FLAG-mCMV-ZsGreen-IRES-Puro vector using NotI/EcoRI restriction sites to generate a TRIM21-overexpression construct. Similarly, TRIM21 DNA was cloned into the pLVX-shRNA2-Puro vector using BamHI/EcoRI restriction sites to create a TRIM21-knockdown construct. Lentiviral plasmids were produced by transfecting 4 × 10^6^ HEK293T cells with 10 μg of either the TRIM21-overexpression construct, TRIM21-knockdown construct, empty pLVX-C-FLAG-mCMV-ZsGreen-IRES-Puro vector, or pLVX-shRNA2-Puro vector. The supernatant was collected after 96 h, filtered through a 0.45 μm membrane, and used to transduce A549 cells. Stably transduced cells were selected using puromycin, and TRIM21 protein levels were assessed by Western blot.

### 2.14 Quantification and statistical analysis

For ELISA assay, the EC_50_ values for serums and mAbs were determined using four-parameter nonlinear regression (GraphPad Prism v10.2.3). For virus neutralizing assay, percent neutralization was calculated as (Sample signals − Virus control signals)/(Blank control signals − Virus control signals) × 100%. Data were fitted using a three-parameter nonlinear regression (GraphPad Prism v10.2.3). For SPR assay, affinity values, including association rates (K_on_), dissociation rates (K_off_), and affinity constants (K_D_), were calculated using Biacore T200 Evaluation Software with 1:1 binding model. A two-tailed Student’s t-test was used when comparing data between two groups, and a one-way analysis of variance (ANOVA) was used for multiple comparisons between more than two groups. Results are expressed as the mean ± SD. Statistical significance was considered at *P < 0.05, **P < 0.01, ***P < 0.001, ****P < 0.0001.

## 3 Results

### 3.1 Isolation of Hexon protein-specific human monoclonal antibodies

**Figure 1:**
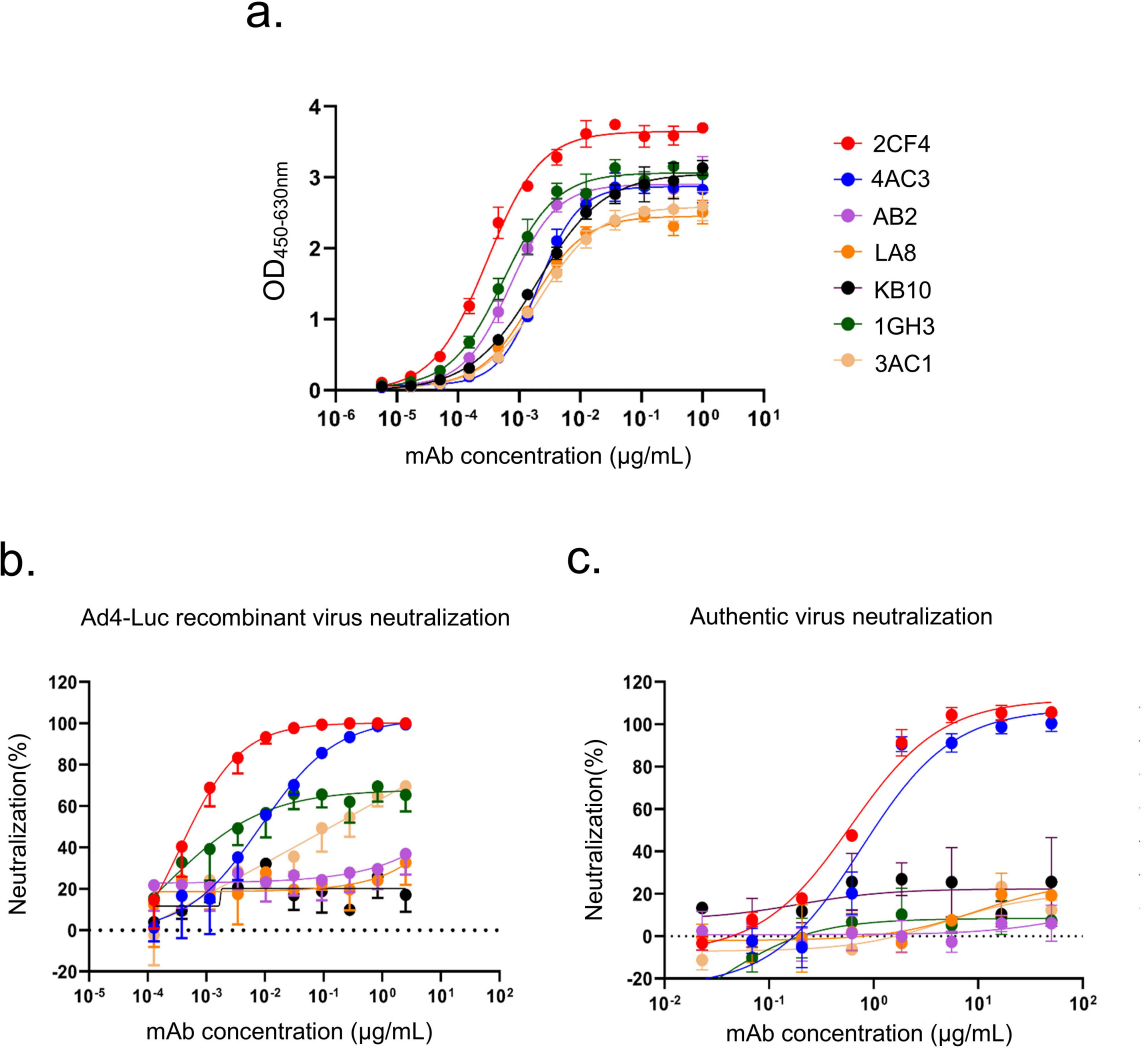
Profiles of binding and neutralization activities of mAbs against HAdV-4. **(a)** Binding curves of all mAbs to the HAdV-4 Hexon protein. **(b)** Neutralization activity of Hexon-reactive mAbs against the Ad4-Luc recombinant virus in A549 cells. **(c)** Neutralization activity of Hexon-reactive mAbs against authentic HAdV-4 in A549 cells. Data are presented as mean ± SD from a representative experiment (n=3).

To isolate (mAbs) and evaluate humoral immune responses to HAdV-4, serum samples were collected from 16 patients. Initial screening focused on PBMCs derived from three convalescent patients who exhibited high levels of neutralization, as determined by serum binding and neutralization assays (**Supplementary Figures S1a, b**). To isolate nAbs with high potency against HAdV-4, we purified the Hexon protein from the Ad4-RI67 strain maintained in our laboratory. Next, to isolate Hexon-specific mAbs, we employed flow cytometry to sort immunoglobulin G–positive (IgG^+^) memory B cells from the PBMCs of convalescent patients, using Hexon as the probe (**Supplementary Figure S1c**). The proportion of Hexon-reactive IgG^+^ B cells ranged from 0.16% to 2.24%, as determined by fluorescence-activated cell sorting (FACS). To further characterize Hexon-specific antibodies, we evaluated the binding specificity of 100 sorted human mAbs using ELISA. Notably, we identified 1, 2, and 4 Hexon-specific mAbs from donors 12, 14, and 15, respectively (**Figure 1a**).

Subsequently, we performed luciferase reporter gene assays for all seven Hexon-binding mAbs, identifying two mAbs that demonstrated neutralizing activity against a replication-competent Ad4-Luc recombinant virus. This virus was constructed by replacing the E3 region with a luciferase expression cassette (**Figure 1b**). The mAbs 2CF4 and 4AC3 exhibited high neutralizing capacity, with half-maximal inhibitory concentrations (IC_50_) of 0.34 ng/mL and 8.54 ng/mL, respectively. To further evaluate the *in vitro* neutralization efficacy of the seven Hexon-specific mAbs against authentic HAdV-4 in A549 cells, we demonstrated that only 2CF4 and 4AC3 exhibited neutralizing activity, while the other mAbs showed no detectable neutralization capacity. These findings were consistent with the neutralization effects observed using the Ad4-Luc recombinant virus system (**Figure 1c**). To characterize the interactions between Hexon proteins and nAbs, we measured the binding kinetics using SPR analysis. The results demonstrated high affinity and strong binding between the Hexon and nAb (**Supplementary Figure S1d**). To determine whether mAbs specifically recognized HAdV-4, we conducted neutralization assays to assess its reactivity against intact HAdV-5 and HAdV-7. Our results confirmed that mAbs specifically targeted intact HAdV-4 (**Supplementary Figure S2**).

Given that the binding affinity, neutralization efficacy and expression level of 4AC3 was significantly lower than that of 2CF4, we selected 2CF4 for further investigation. Under heating denatured conditions, mAb 2CF4 failed to bind the target antigen in ELISA, whereas it exhibited strong binding to native antigen (**Supplementary Figure S3**). This indicates that 2CF4 recognizes a conformation-dependent epitope that is disrupted upon protein denaturation.

### 3.2 Characterization of Hexon protein–specific human mAbs

**Figure 2:**
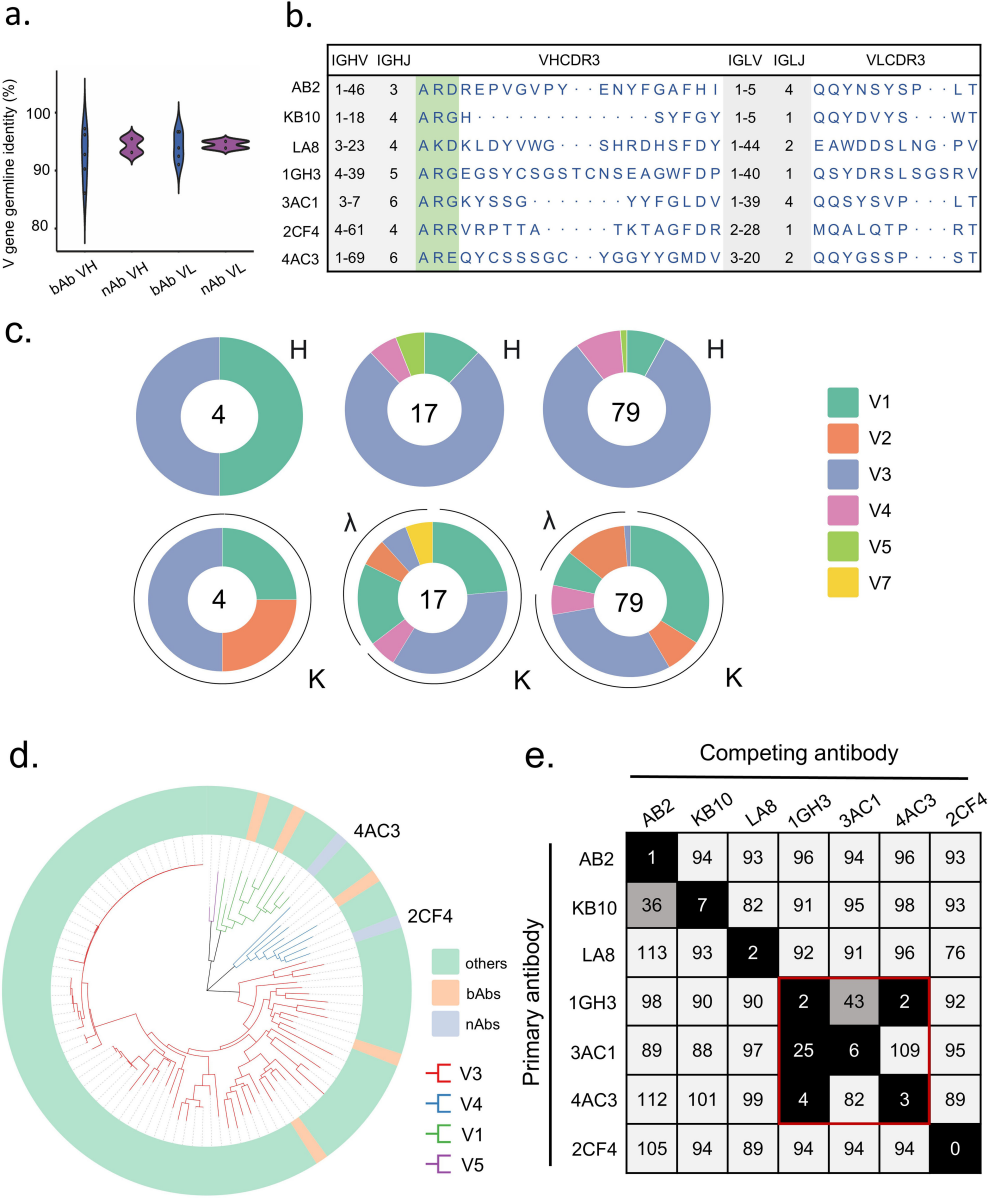
Binding properties of Hexon protein-specific mAbs. **(a)** The VH and VL gene identities from the germlines of nAbs and bAbs. **(b)** The VH and VL gene usage, J gene usage, and CDR3 aa sequences of mAbs. **(c)** Distribution of V gene families in heavy and light chains of all distinct clones (the total number is shown in the center of the pie charts) for each donor. **(d)** Phylogenetic trees of all the sort mAbs. **(e)** The numbers in the box indicate the percentage of binding of the competing mAbs following the binding by primary antibody. The mAbs were considered competing if the inhibition percentage was <33% (black boxes with white numbers). The mAbs were deemed to be non-competing for the same site if the percentage was >66% (white boxes with black numbers). The grey boxes with black numbers indicate an intermediate phenotype (33 to 66%).

NAbs exhibited a higher average germline identity for the heavy chain variable region (VH) at 94.3%, compared to binding antibodies (bAbs), which showed an average of 92.5%. In contrast, the light chain variable region (VL) displayed comparable levels of somatic hypermutation (SHM), with germline identities of 94.5% for nAbs and 94.2% for bAbs (**Figure 2a**). The lengths of the complementarity-determining region 3 (CDR3) varied among donors, with VH CDR3 lengths ranging from 9 to 22 amino acids and VL CDR3 lengths ranging from 9 to 12 amino acids (**Figure 2b**). The distribution of sequenced light chain (IgL) VH3 gene families were consistent across the three donors, with this gene family being the most frequently utilized. However, variability was observed in the preferences for heavy chain (IgH) gene families among different donors (**Figure 2c**).

To characterize the diversity in gene usage and affinity maturation, phylogenetic trees of these Hexon-specific mAbs were constructed based on the amino acid sequences of VHDJH using the neighbor-joining method in Chiplot. The results revealed considerable diversity in VH gene usage among the 100 mAbs derived from the three donors, with the VH3 germline gene being the most frequently utilized. (**Figure 2d**).

To investigate potential overlapping antigenic sites among different mAbs, we performed a competition-binding assay using ELISA with several representative mAbs. Among these, 1GH3 competed with 4AC3 and 3AC1, indicating that these three mAbs recognize shared epitopes on Hexon, each of the remaining antibodies targets a unique epitope. These findings suggest that the antibody responses elicited by natural HAdV-4 infection exhibit significant diversity in epitope recognition of Hexon proteins (**Figure 2e**).

### 3.3 2CF4 protect against HAdV-4 challenge in the Stat1^-/-^mouse model

First, we utilized BALB/c mice to evaluate the *in vivo* efficacy of anti-HAdV-4 nAb. BALB/c mice (6–8 weeks old) were inoculated with the Ad4-Luc recombinant virus and intraperitoneally injected with PBS, 10 μg of 2CF4, or 2 μg of 2CF4 one day before viral challenge. Luciferase activity in the mice was monitored using bioluminescence imaging at 3 h post-challenge and for five consecutive days. The results showed that luciferase activity was completely undetectable in the 10 μg 2CF4 group at 3 h post-challenge, while it peaked in the virus-only group. No viral rebound was observed during the five-day monitoring period, indicating that the antibody treatment completely neutralized the virus (**Supplementary Figure S4**). However, after 14 consecutive days of observation, no mortality was observed in the PBS group, highlighting the urgent need to establish a lethal animal model for HAdV-4.

**Figure 3:**
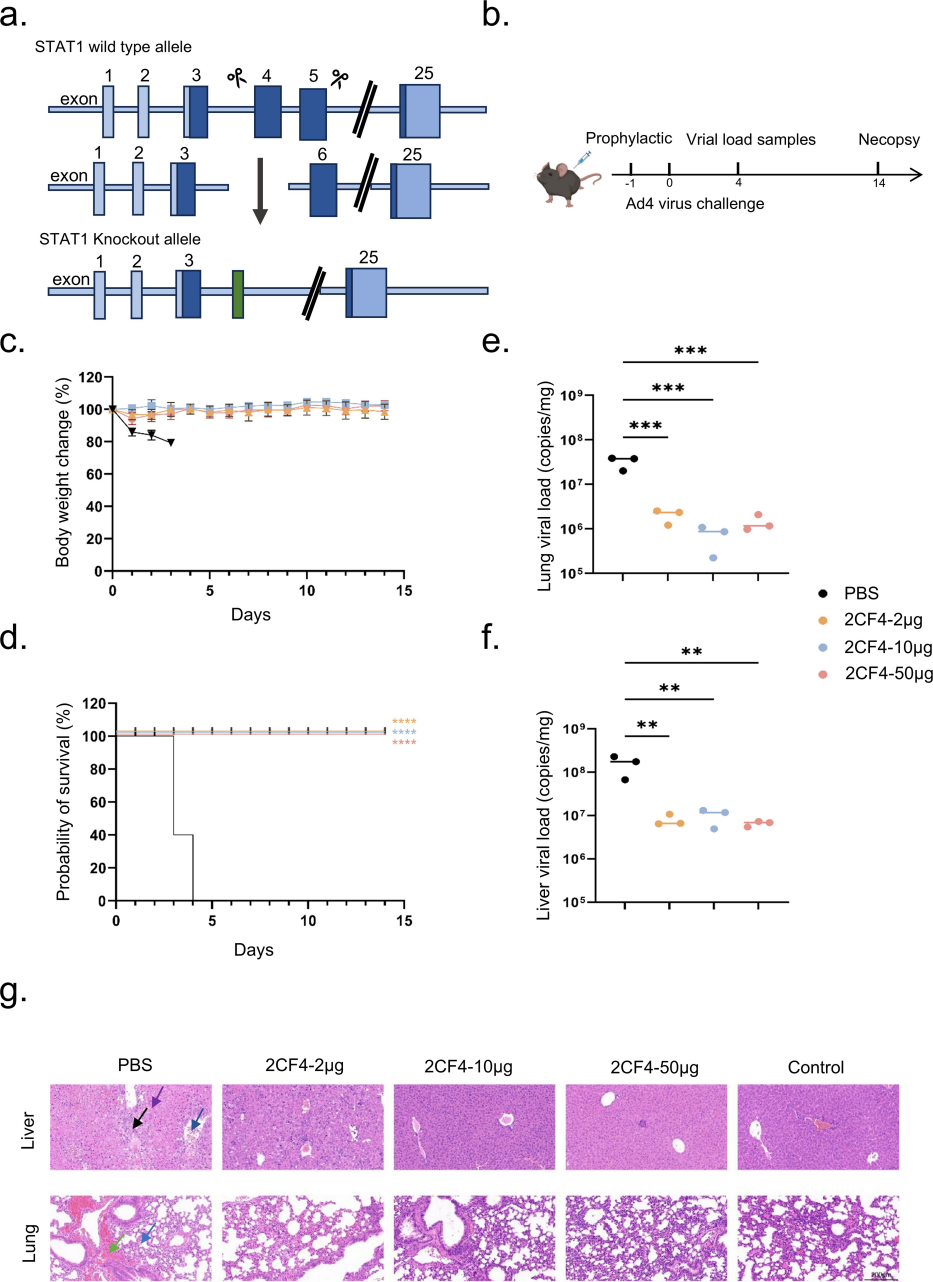
Prophylactic efficacy of 2CF4 in the Stat1^-/-^ transgenic mouse model of HAdV-4 infection. **(a)** Schematic illustration of the principle of CRISPR/Cas9 technology to obtain Stat1 knockout. **(b)** Prophylactic study schema. **(c)** Body weight changes of mice in the prophylactic groups. The mean ± SD are shown (n = 5). **(d)** Survival rates of Stat1^-/-^ transgenic mice (n = 5) in the prophylactic groups and PBS in mice pre-treated with mAb 2CF4 at 50μg, 10μg and 2μg. Statistical analysis was assessed using Kaplan-Meier method, with ***P < 0.001, ****P < 0.0001. **(e)** Virus copy numbers in the lungs on day 4. Statistical analysis was assessed using a one-way analysis of variance (ANOVA), with ***P < 0.001. **(f)** Virus copy numbers in the liver on day 4. Statistical analysis was assessed using a one-way analysis of variance (ANOVA), with **P < 0.01. **(g)** Histopathological changes in the lungs and livers of HAdV-4 infected mice collected at day 4. In liver tissue sections, black arrows represent a small amount of connective tissue proliferation, purple arrows represent a large number of hepatocytes with scattered necrosis, and blue arrows represent a small number of hepatocytes with steatosis. In lung tissue sections, blue arrows represent small amounts of granulocyte infiltration seen in the alveolar walls, and green leads represent focal hemorrhages seen in the lung tissue. Control represents the uninfected control. The magnification is 20x. Scale bars, 200 μm.

**Figure 4:**
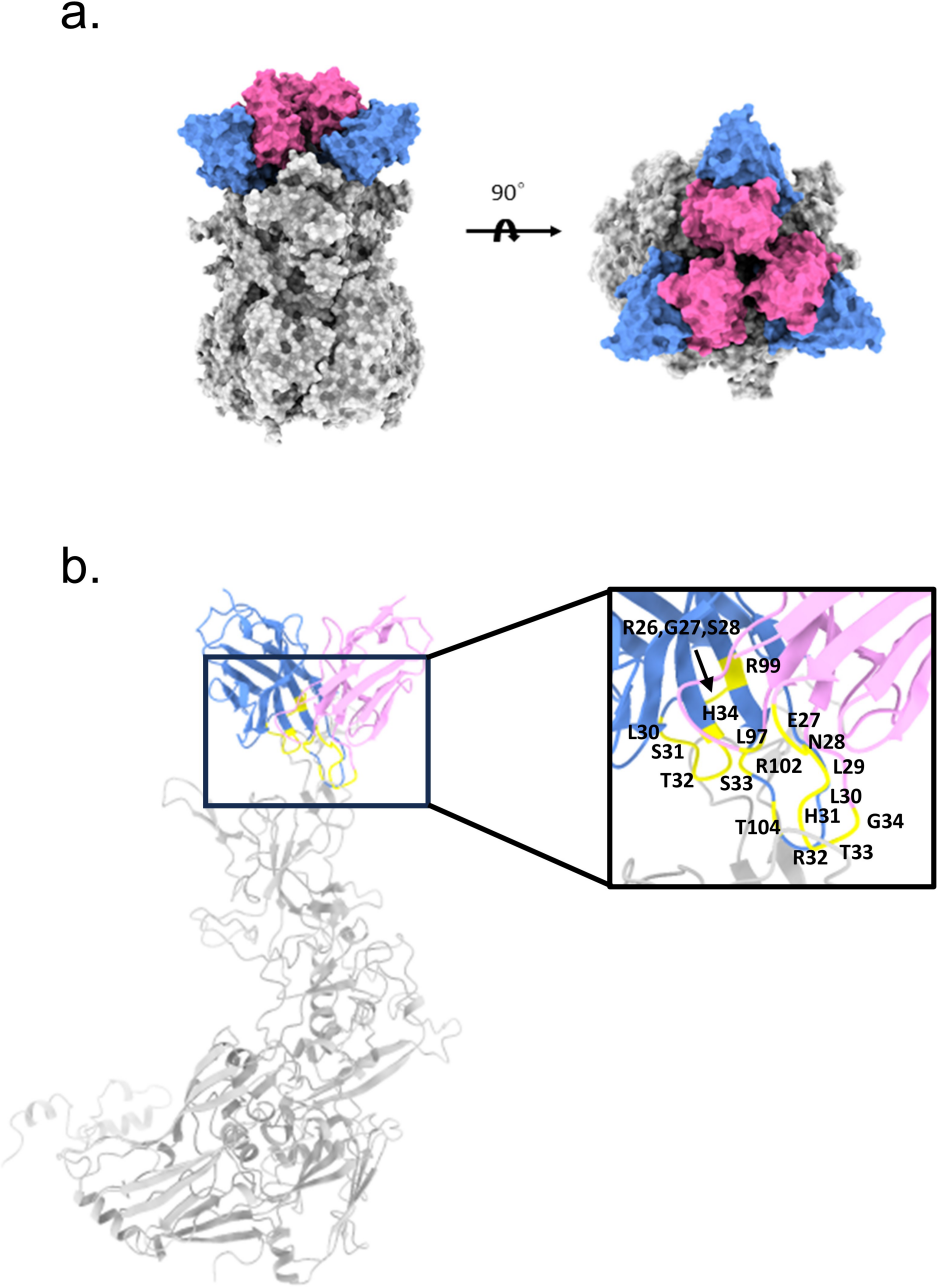
Structural prediction of Hexon and mAb 2CF4 complexes **(a)** The domain-colored structures of the Hexon protein in complex with nAb 2CF4, viewed along two perpendicular orientations. The heavy and light chains of 2CF4 are colored blue and pink, respectively. The Hexon protein are colored gray, neutralizing antibodies against Hexon included HAdV-4. **(b)** Binding mode of the antibody-antigen interaction surface. The mutation sites on the antibody are marked in yellow.

We employed Stat1^-/-^ transgenic mice to assess the *in vivo* efficacy of anti HAdV-4 nAb based on previously established methodologies (34) (**Figure 3a**). Stat1^-/-^ transgenic mice (6–8 weeks old) were inoculated with 3 × 10^10^ PFUs of authentic HAdV-4 (**Figure 3b**). All mice in the PBS group succumbed to the infection within four days post-challenge, accompanied by significant weight loss. In contrast, mice in the prophylactic group, which received mAb 2CF4 at doses of 50 μg (≈2.5 mg/kg), 10 μg (≈0.5 mg/kg), or 2 μg (≈0.1 mg/kg) one day prior to infection, survived the lethal challenge without noticeable weight loss (**Figures 3c, d**). Furthermore, mice treated with 2CF4 exhibited significant reductions in viral copy numbers in both lung (**Figure 3e**) and liver tissues (**Figure 3f**), along with marked improvements in lung and liver pathological tissues damage induced by the viral infection (**Figure 4g**). There were no significant differences in pathological sections of the heart, spleen and kidney between the PBS group and the antibody treatment group (**Supplementary Figure S5**). These findings demonstrate that mAb 2CF4 can confer protective effects even at low dose concentrations.

### 3.4 Structural prediction of nAbs in complex with Hexon protein

To elucidate the interactions between mAb 2CF4 and the Hexon protein, we employed AlphaFold2-multimer to predict the structure of the antigen-antibody complex (**Figure 4a**). Following the structural simulation analysis of the antigen-antibody complex, we aimed to identify the key residues mediating the interactions between Hexon and 2CF4 through a systematic structure-guided mutagenesis approach. We selected a total of 20 amino acid residues from the heavy and light chains of mAb 2CF4, including combinatorial mutations in heavy chain complementarity determining region 3 (HCDR3) (**Figure 4b**).

Subsequently, we evaluated the binding and neutralization activities of the resulting mutants using ELISA and neutralization assays. These experiments allowed us to quantitatively assess the impact of the mutations on the formation of the Hexon-2CF4 complex. By systematically analyzing the binding affinities and neutralization efficiencies of each mutant, we identified specific amino acids essential for optimal interaction and functional efficacy of mAb 2CF4. The results revealed that certain mutations significantly impaired both binding and neutralization, highlighting their critical role in the antibody’s mechanism of action. This study not only identifies key interaction residues but also enhances our understanding of the molecular mechanisms underlying the efficacy of mAb 2CF4 against Hexon, providing valuable insights for the design of more effective therapeutic antibodies targeting similar viral proteins.

### 3.5 Residues critical for the binding of 2CF4

To identify the key residues involved in the interactions between Hexon and mAb 2CF4, we utilized a structure-guided mutagenesis approach. This analysis identified a total of 20 key amino acid residues located in the antibody that may play an important role in the binding affinity and specificity of 2CF4. These residues are located in the H chain of the antibody, specifically: R26, G27, S28, S30, S31, T32, S33, H34, R99, R102, and T104. Additionally, residues from the κ chain were also identified, including E27, N28, L29, L30, H31, R32, T33, G34, and L97.

**Figure 5:**
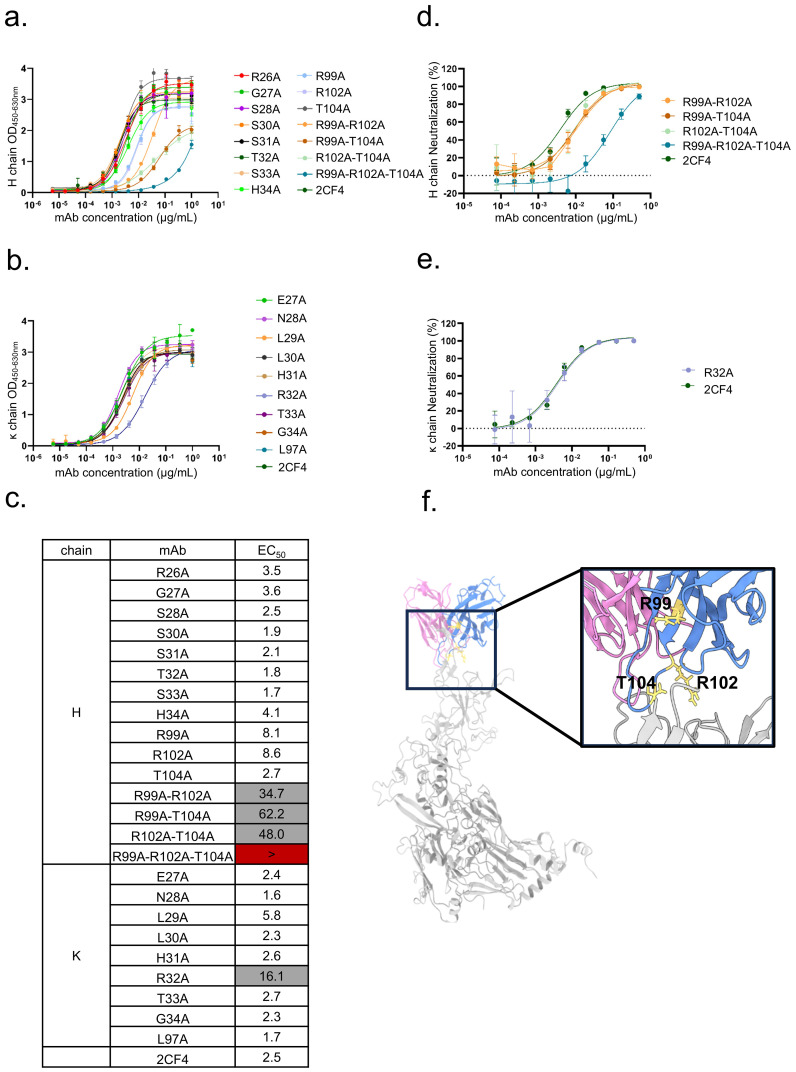
Validation of key residues located at the Hexon-2CF4 interfaces. **(a)** The binding activities of the H chain mutant and WT-2CF4 were determined by ELISA. **(b)** The binding activities of the k chain mutant and WT-2CF4 were determined by ELISA. **(c)** Heatmap showing the binding of mAbs to Hexon proteins determined by using ELISA. The EC_50_ value for each Hexon-mAb combination is shown, with white shading, grey, or dark red indicating high, low, or no detectable binding, respectively. EC_50_ values greater than 10,000 ng/mL are indicated (>). The neutralization activities of the mutant and WT-2CF4. **(d)** Neutralization of the H chain mutant and WT-2CF4 to Ad4-Luc recombinant virus in A549 cells. **(e)** Neutralization of the K chain mutant and WT-2CF4 to Ad4-Luc recombinant virus in A549 cells. All Data were shown as mean ± SD of a representative experiment (n=3). **(f)** R99, R102, T104 on HCDR3 may be critical amino acids.

The resulting mutants were subjected to a series of assays, including ELISA and neutralization assays, to thoroughly evaluate their interactions with Hexon and their efficacy in neutralizing the virus. Binding assays revealed a significant reduction in binding activity when the HCDR3 residues (R99, R102, T104) were simultaneously mutated (**Figures 5a-e**). This finding highlights the critical role of HCDR3 in the recognition and binding of Hexon by mAb 2CF4.

Furthermore, the mutation of κ chain residue R32 also diminished binding activity, underscoring its contribution to the interaction network. Notably, neutralization assays demonstrated that the reduction in neutralization activity was particularly pronounced in mutants with combinatorial mutations in the HCDR3 (R99, R102, T104) (**Figure 5f**). This suggests that these specific residues are essential for the neutralizing capacity of mAb 2CF4 against HAdV-4, emphasizing their importance in the design of effective therapeutic antibodies targeting viral infections. Collectively, these results provide valuable insights into the molecular mechanisms underlying the antibody’s interaction with Hexon and the structural determinants critical for its neutralizing function.

### 3.6 TRIM21 mediates intracellular antibody neutralization

**Figure 6:**
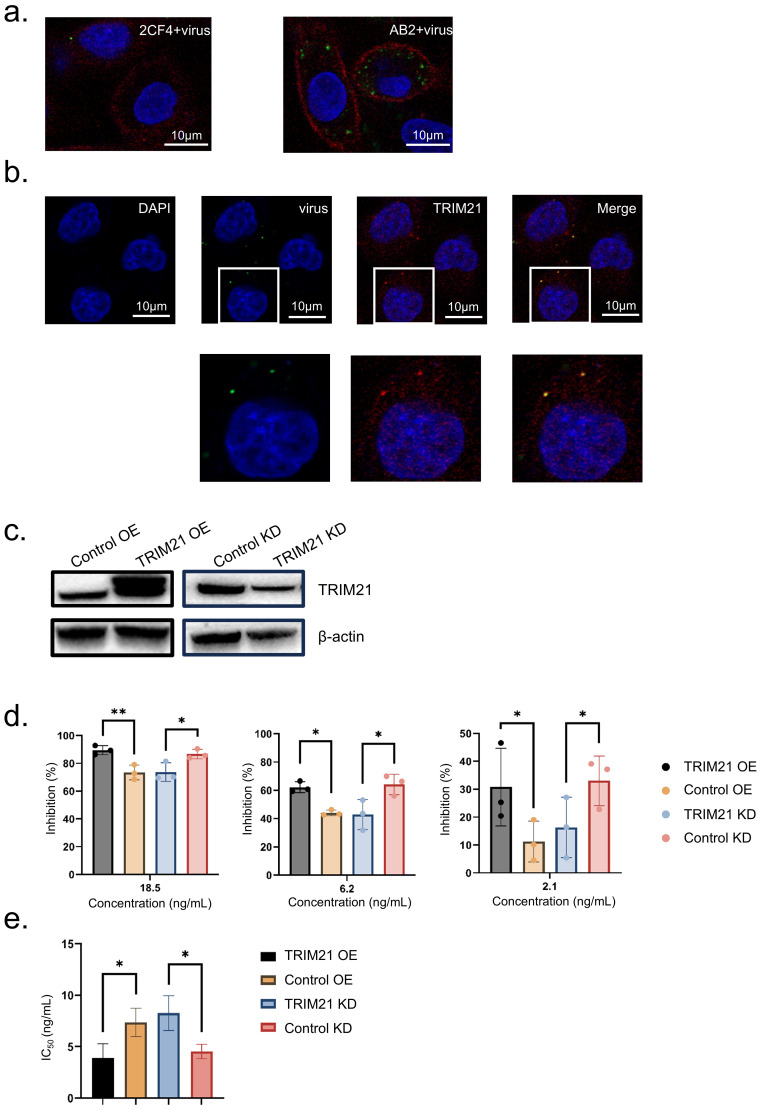
TRIM21 mediates intracellular antibody neutralization. **(a)** Confocal microscopy images to detect intracellular fluorescence. Scale bars, 10 μm. **(b)** Confocal microscopy images of adenovirus-infected A549 cells. Adenovirus precoated in antibody and detected after infection with an Alexa Fluor 488 secondary (green) can be seen inside cells. Scale bars, 10 μm. **(c)** Western blot of TRIM21 protein levels in each condition. **(d)** At concentrations of 18.6, 6.2, and 2.1 ng/mL, the inhibition rate data were shown as mean ± SD of three repetitive experiments. Statistical analysis was assessed using a two-tailed Student’s t-test, with *P < 0.05, **P < 0.01. **(e)** IC_50_ Data were shown as mean ± SD of three repetitive experiments. Statistical analysis was assessed using a two-tailed Student’s t-test, with *P < 0.05.

To investigate the mechanism of antibody-mediated neutralization against HAdV-4, we performed immunofluorescence to analyze A549 cells infected with a pre-incubated mixture of the mAb 2CF4 and virus. The control group consisted of a mixture of the conjugated antibody AB2 and the virus. 6 h post-infection, the cells were fixed, permeabilized, and stained with a fluorescent anti-IgG antibody to detect antibody-bound viral particles. Using confocal microscopy to observe intracellular fluorescence, we found that viral particles entered the cells together with antibody binding. Notably, the number of viral particles in the neutralized antibody-virus mixture was significantly reduced compared to the conjugated antibody-virus mixture. These results indicate that antibodies can enter cells and exert intracellular neutralization mechanisms following viral binding (**Figure 6a**).

TRIM21 acts as a high-affinity receptor for the Fc region of antibodies (IgG) intracellular, to determine whether antibody-bound viral particles interact with cytoplasmic TRIM21 after cellular entry, we performed co-staining experiments. These experiments revealed the colocalization of TRIM21 with neutralizing antibody-bound virions, suggesting a potential role for TRIM21 in intracellular neutralization (**Figure 6b**).

To further investigate the role of TRIM21 in the intracellular neutralization mechanism, we generated stable TRIM21 overexpression and knockdown cell lines. Confirming the expression levels using Western blot analysis, thereby validating our experimental model. (**Figure 6c**). We then used the Ad4-Luc recombinant virus to assess infection efficiency through neutralization assays. Overexpression of TRIM21 enhances antibody-mediated neutralization activity, while knockdown of TRIM21 reduces this neutralizing effect. The results indicate that TRIM21 plays a role in intracellular neutralization. (**Figure 6d**). Analysis of the IC_50_ values revealed a significant enhancement in neutralization efficacy following TRIM21 overexpression (**Figure 6e**). These findings indicate that TRIM21 plays a critical role in mediating adenovirus neutralization, highlighting its importance in the intracellular immune response against viral pathogens.

## 4 Discussion

HAdV-4 is a notable pathogen associated with acute respiratory infections (ARIs), exhibiting a predominantly sporadic pattern while also capable of causing localized epidemics or large-scale outbreaks, some of which have resulted in fatalities. Although a vaccine against HAdV-4 has been developed, its use has been largely restricted to military recruits, leaving a significant gap in preventive measures for other regions and populations, particularly in China, where no vaccine or safe and effective antiviral therapy is currently available (31). Consequently, there is an urgent need to prioritize the exploration of effective antiviral agents against HAdV-4. In this context, we successful generation of high-potent nAbs lays the foundation for developing treatments to combat HAdV-4 associated severe respiratory diseases. Our study demonstrates that naturally occurring human HAdV-4 mAbs, isolated from the B cells of three convalescent donors, exhibit diversity in gene usage and epitope recognition of the Hexon protein. Currently, available anti-HAdV-4 antibodies are limited to mouse anti-humanized versions, with no fully human monoclonal antibodies yet developed. Through a systematic screening process, we identified seven strains of antibodies with significant binding activity, among which 2CF4 and 4AC3 were shown to effective neutralization capabilities to the Hexon protein of HAdV-4. Compared with the Ad4-Luc recombinant virus, the observed higher neutralization IC_50_ value of the authentic virus may be attributed to the reduced pathogenicity of the recombinant virus due to the lack of the E3 gene, or the 100 TCID_50_ detection value being higher than the actual value. The identification of these antibodies not only enhances our understanding of the humoral immune response to HAdV-4 but also opens avenues for the potential development of targeted immunotherapies. Further studies will be essential to evaluate the therapeutic efficacy and safety of these antibodies in clinical settings, offering hope for improved management of HAdV-4-associated respiratory infections.

It is well established that HAdV mAbs are typically serotype-specific, with previous studies indicating minimal or no cross-reactivity with other HAdV species (35). To validate this assertion, we conducted cross-neutralization assays with HAdV-5 and HAdV-7, which belong to different species. Our results confirmed that the mAbs 2CF4 and 4AC3 exclusively recognize HAdV-4, demonstrating their specificity. Due to its superior binding affinity, neutralization efficacy, and expression levels compared to 4AC3, 2CF4 was selected for further investigation.

Prior to this study, there has been a limited number of nAbs against HAdV demonstrated to confer protection *in vivo*, likely due to the scarcity of suitable animal models that effectively mimic HAdV infection and pathogenesis. The signal transducer and activator of transcription STAT1 protein is pivotal in orchestrating the immune response against viral and other pathogenic infections. As a transcription factor, STAT1 activates the expression of numerous genes, many of which are well-documented for their antiviral properties (32). Previous studies have shown that tyrosine-phosphorylated STAT1 is selectively targeted by a distinct mechanism in adenovirus-infected cells (36). Our study employs Stat1^-/-^ transgenic mice, providing a robust framework for elucidating the *in vivo* protective effects of anti-HAdV-4 nAb. In these Stat1^-/-^ transgenic mice, we observed that the inhibitory effect of the nAb closely correlated with its respective *in vitro* neutralizing activity, thereby establishing a link between *in vitro* neutralization potency and *in vivo* protective efficacy. Although our data robustly support the prophylactic efficacy of mAb 2CF4 against HAdV-4 in Stat1^-/-^ transgenic mice, we recognize that the therapeutic potential remains to be evaluated. The robust *in vitro* neutralizing activity of mAb 2CF4 demonstrates therapeutic potential. However, confirmation of *in vivo* efficacy awaits validation in rigorous post-exposure treatment models. In addition to assessing protective efficacy, a comprehensive evaluation of viral kinetic parameters, such as viral load decay rates, antibody persistence duration, and the risk of viral rebound after antibody elimination will be indispensable for a thorough characterization of its therapeutic profile.

To further elucidate the interaction between mAb 2CF4 and the Hexon protein of HAdV-4, we employed a computational approach to identify the binding site, complemented by experimental techniques involving targeted mutagenesis. Through prediction of antigen-antibody complexes, we developed a model of the mAb 2CF4-Hexon interaction, which indicated that the residues R99, R102, and T104 play critical roles in this process. Indeed, site-directed mutagenesis of the antigen would provide definitive validation of the neutralizing epitopes. However, since our current Hexon protein was purified from wild-type virions rather than recombinant expression systems, this method is not feasible. In the future, we will establish prokaryotic and eukaryotic expression systems to produce recombinant Hexon protein, which will enable precise mutagenesis studies for subsequent epitope mapping.

In this context, adaptive immunity, represented by antibodies, provides targeted pathogen recognition, while neutralization is mediated by the intracellular receptor TRIM21 and an innate degradation pathway. This interplay of mechanisms could impose significant constraints on the ability of viruses to evolve and evade immune responses (37). We conducted immunofluorescence assays to monitor intracellular fluorescence and observed that the neutralizing activity of antibodies occurs intracellularly. A subset of viral particles colocalized with TRIM21 within the cells, suggesting that TRIM21 is involved in the intracellular neutralization mechanism.

In conclusion, mAbs 2CF4 and 4AC3 demonstrated the highest affinity for HAdV-4 among the antibodies tested, exhibiting a pronounced capacity to inhibit HAdV-4 infection at low concentrations *in vitro*. MAb 2CF4 effectively neutralizes HAdV-4 *in vivo*, highlighting its potential for prophylactic applications. Future studies will focus on further validating the protective efficacy of 2CF4 *in vivo*. Given the rising incidence of adenovirus infections and the current lack of effective treatment options, the development of mAb 2CF4 represents a promising advancement in the fight against this virus.

## Data Availability

The original contributions presented in the study are included in the article/**Supplementary Material**. Further inquiries can be directed to the corresponding author.
