# Supplementary material for: Protection against lethal HAdV-4 challenge in STAT1 mice by novel human monoclonal antibodies
Source: Front Immunol. 2025 Jun 19;16:1613945. doi: 10.3389/fimmu.2025.1613945 (PMC12221930; doi:10.3389/fimmu.2025.1613945)
Supplement: Supplementary file 1 [file Table1.docx]

Supplementary Material

## Supplementary Figure 1


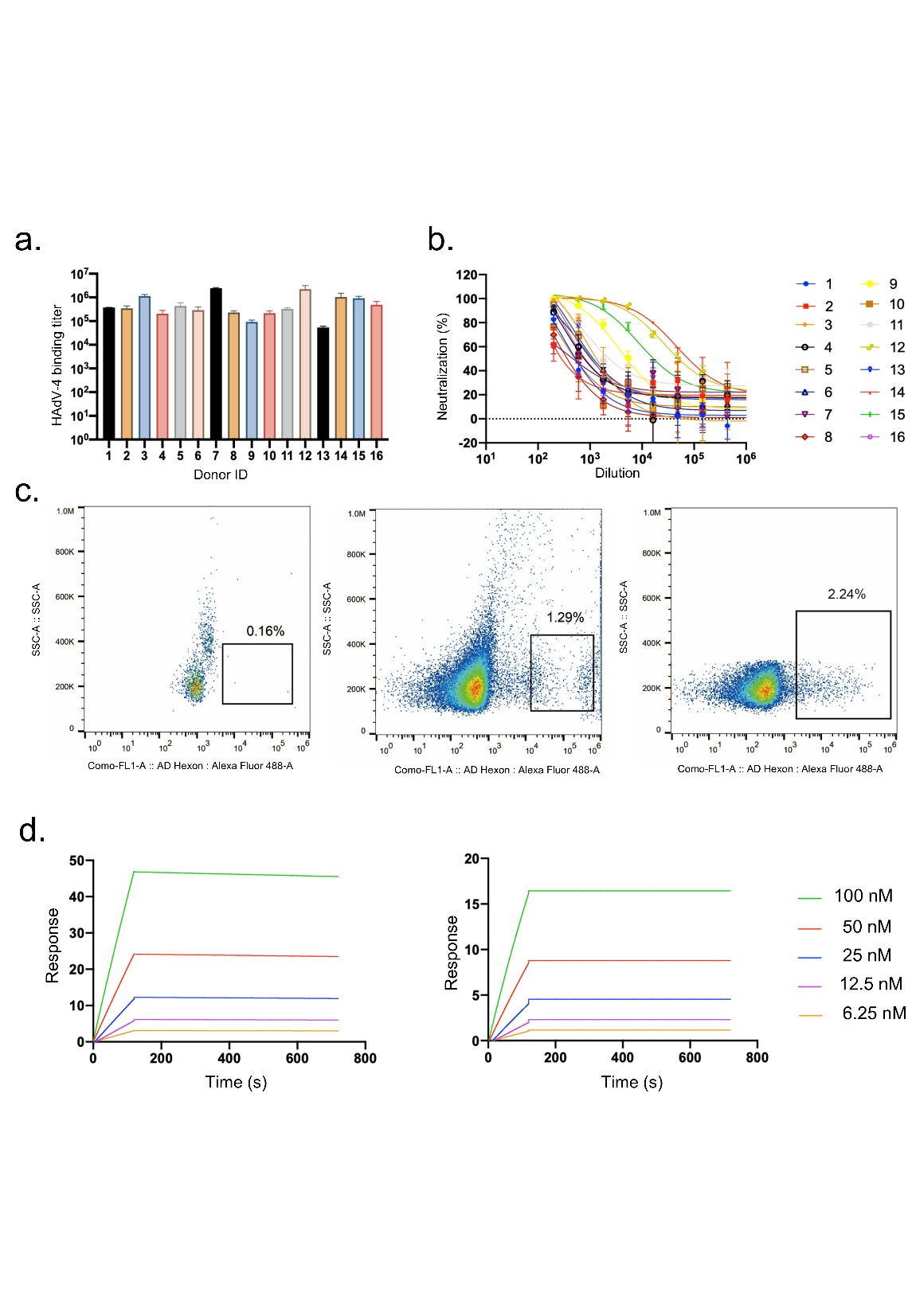


Figure S1. Isolation of antigen-specific mAbs from convalescent patients of HAdV-4. (a) Reactions of serum to HAdV-4 proteins. Hexon protein was used in ELISA to test the binding of serum. Serum of heathy donors were used as control, and cut-off values were calculated as optical density (OD) 450 of control × 2.1. Data were shown with mean ± SD of a representative experiment (n=3). (b) Ad4-Luc recombinant virus neutralization IC_50_ titers against HAdV-4 variants in serum samples. Virus neutralization IC_50_ titers against HAdV-4 in serum samples from convalescent patients. Data were shown with mean ± SD of a representative experiment (n=3) (c) CD3^−^CD19^+^IgG^+^CD27^+^Hexon^+^ cells were isolated from blood after infection and single cells were sorted to isolate antibody-secreting cells. (d) SPR sensorgrams of mAbs binding to Hexon proteins.

## Supplementary Figure 2


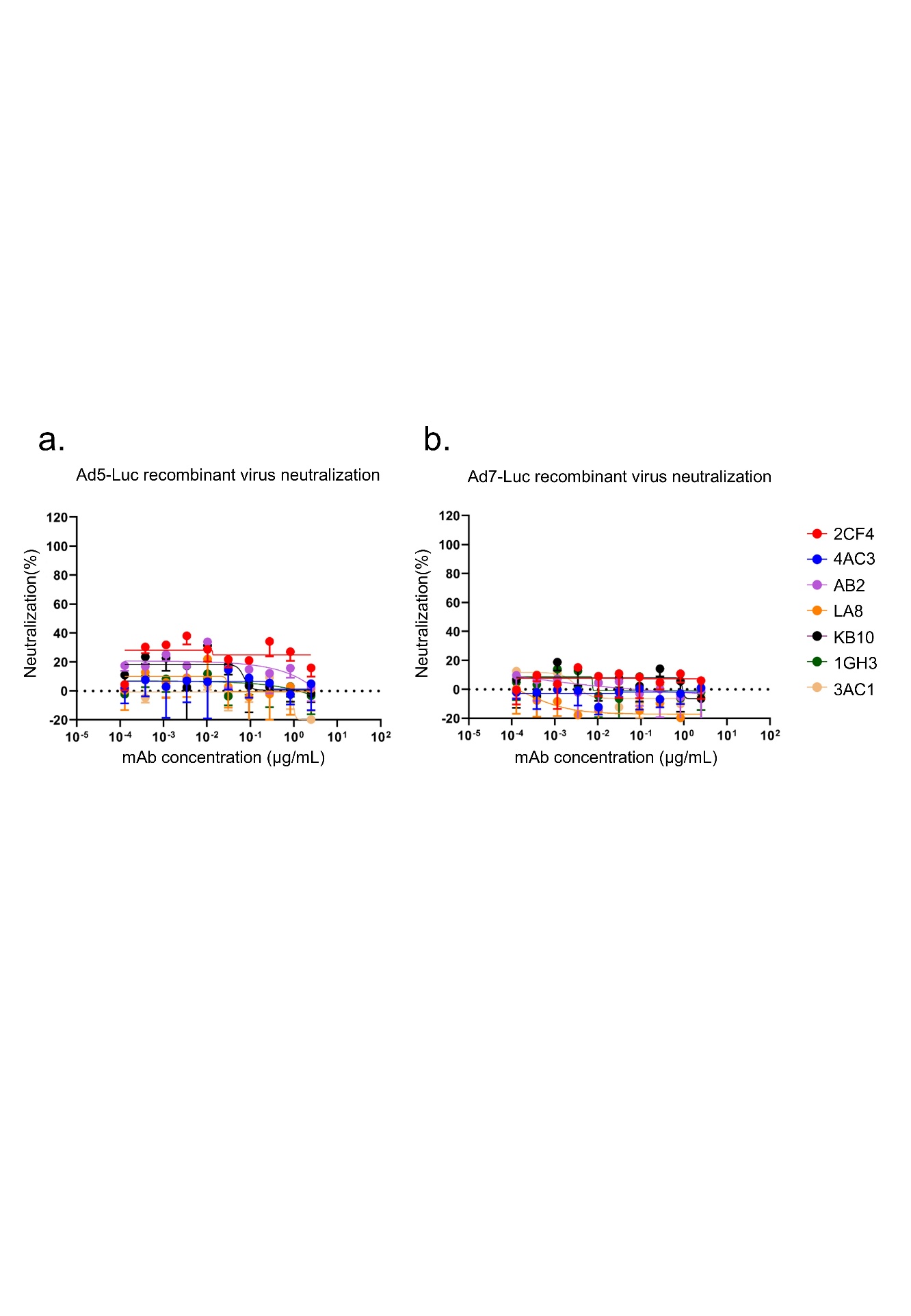


Figure S2. mAbs cross-react with several types of HAdV. (a) Neutralization of Hexon-reactive mAbs against Ad5-Luc virus in A549 cells. (b) Neutralization of Hexon-reactive mAbs against Ad7-Luc virus in A549 cells. All data were shown as mean ± SD of a representative experiment (n=3).

## Supplementary Figure 3


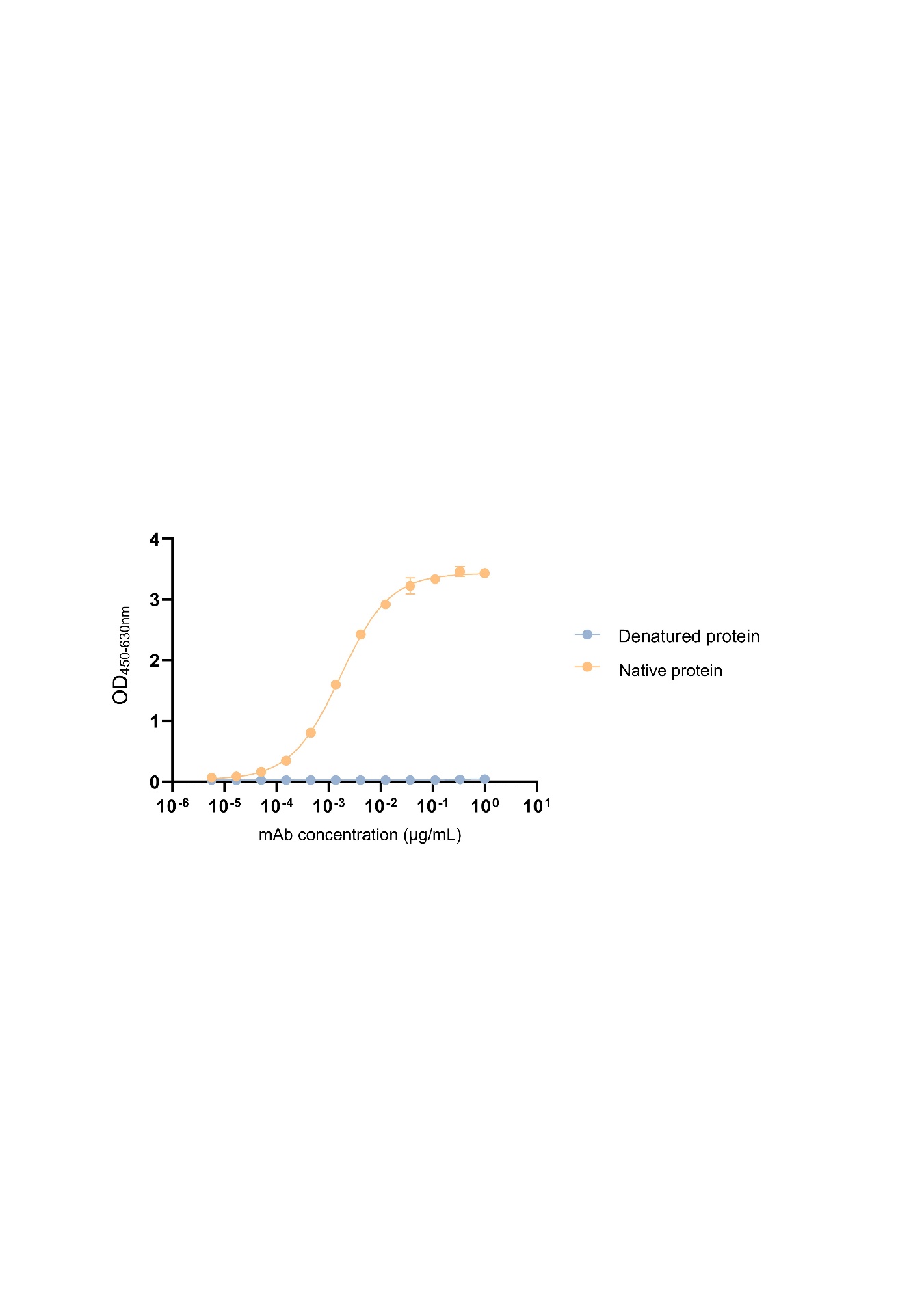


Figure S3. Conformational epitope validation of mAb 2CF4 through antigen denaturation assays. Data were shown with mean ± SD of a representative experiment (n=3).

## Supplementary Figure 4


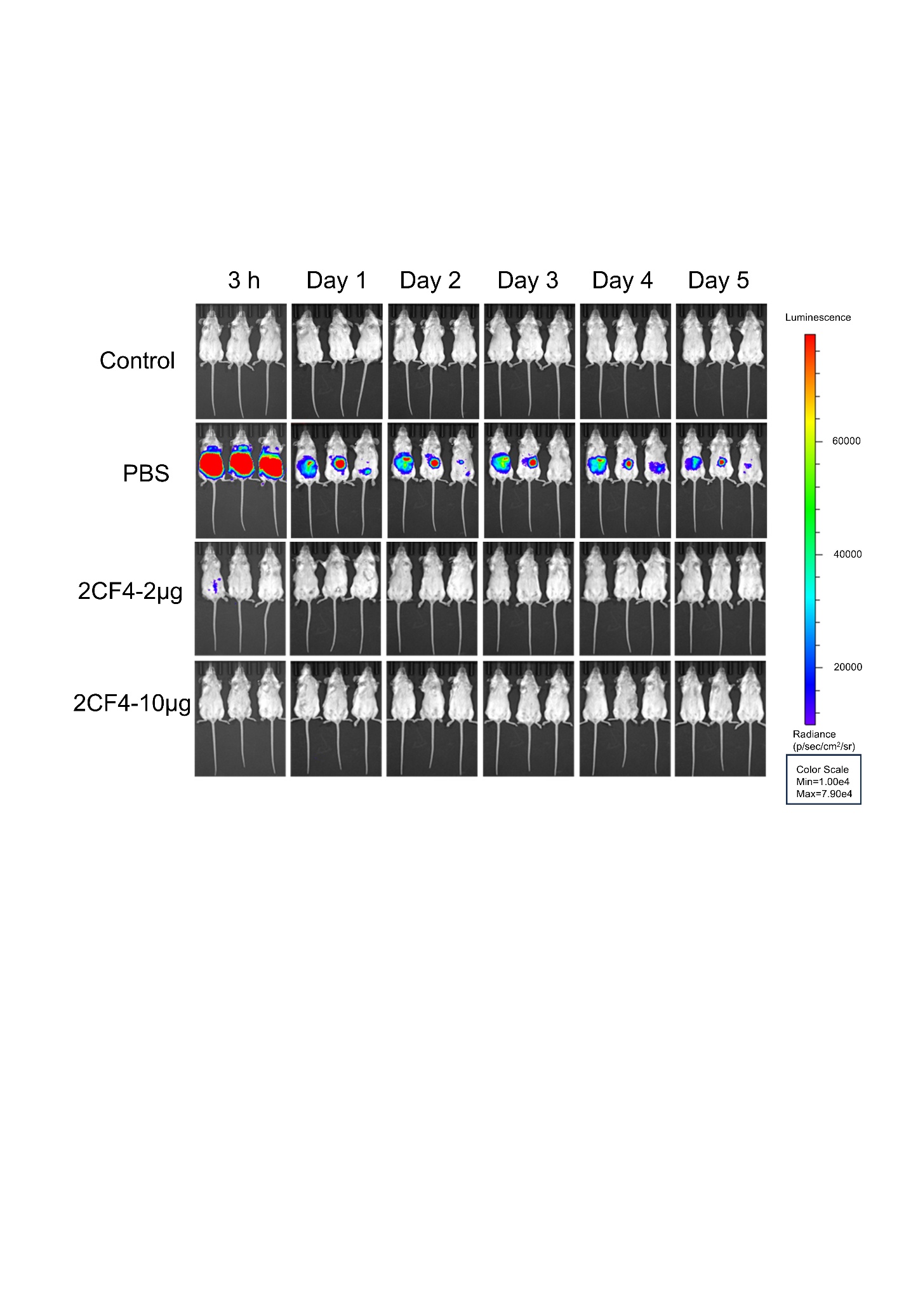


Figure S4. Bioluminescence imaging for detection of luciferase expression in virally repopulated BALB/c mice. Prophylactic groups of mice (n=3) were pretreated with PBS, 10 μg mAb 2CF4, or 2 μg mAb 2CF4 prior to viral challenge, along with an untreated control group.

## Supplementary Figure 5


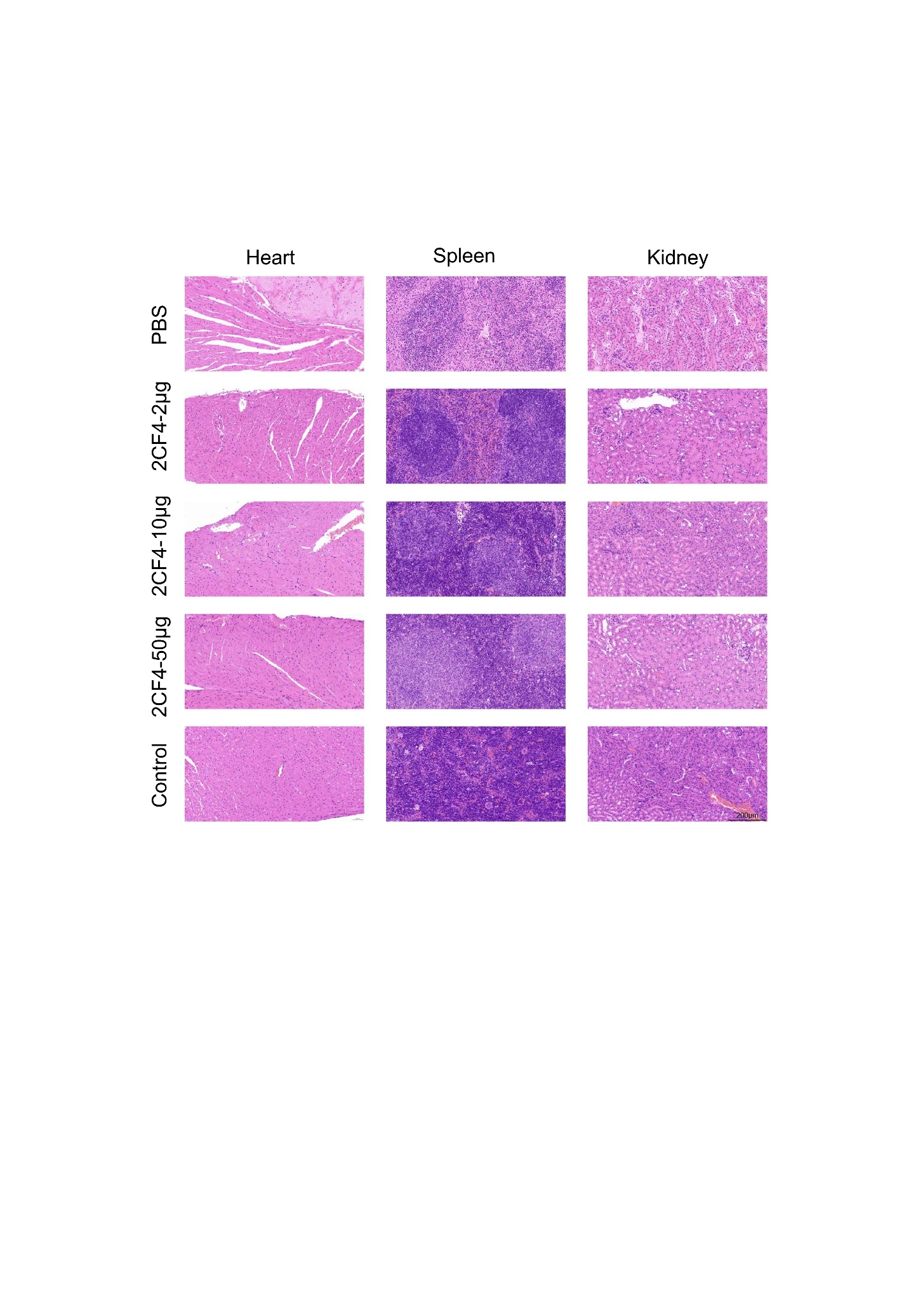


Figure S5. Pathologic sections of the heart, spleen and kidney four days after the attack of Stat1^-/-^transgenic mice. The magnification is 20×. Scale bars, 200 μm.
